# Selection and validation of reference genes for quantitative real-time PCR analysis of gene expression in *Cichorium intybus*

**DOI:** 10.3389/fpls.2015.00651

**Published:** 2015-08-18

**Authors:** Marianne Delporte, Guillaume Legrand, Jean-Louis Hilbert, David Gagneul

**Affiliations:** Université Lille, INRA, ISA, Université d’Artois, Université du Littoral Côte d’Opale, EA 7394 – ICV – Institut Charles ViolletteLille, France

**Keywords:** gene expression, normalization, cell culture, specialized metabolism, phenylpropanoids, methyljasmonate

## Abstract

Plant polyphenols represent a huge reservoir of bioactive compounds. Industrial chicory, an important crop from northwestern Europe, accumulates an original combination of such compounds, i.e., chlorogenic, isochlorogenic, caftaric, and chicoric acids arising from the phenylpropanoid pathway. For a complete understanding of these biochemical pathways, analyses of gene expression using quantitative real-time PCR (qRT-PCR) should be considered. Because cell cultures are a model of choice for specialized metabolism investigations, this study described for the first time the validation of reference genes for this system in chicory. Eighteen potential reference genes were obtained by mining expressed sequence tag databases of chicory for orthologs of *Arabidopsis thaliana* genes currently used as reference genes. Twelve genes passed the qRT-PCR standard requirements and their expression stability across different samples was tested using three distinct softwares: geNorm, NormFinder, and BestKeeper. In cell cultures grown under various conditions, *TIP41* (TIP41 like protein) was shown to be the most stable gene. Further validation of the proposed reference genes was done by normalization of expression levels of a group of genes of interest. In order to assess the potentiality of the proposed list of candidate reference genes, theses genes were in parallel tested on another experimental design, i.e., chicory seedlings. In this case, the best reference gene identified was *Clath* (Clathrin adaptator complex subunit). The results highlight the importance of the use of properly validated reference genes to achieve relevant interpretation of qRT-PCR analyses. Here, we provide a list of reference genes suitable for future gene expression studies in chicory.

## Introduction

Plant specialized metabolites, also known as secondary metabolites in opposition to so called primary metabolites, represent a huge reserve of bioactive compounds amenable for a wide range of human applications. Among them, polyphenols are particularly desirable in food crops due to their numerous health benefits notably because of their antioxidant properties (Scalbert et al., 2005). Industrial chicory (*Cichorium intybus* L. var *sativum*) is a member of the *Asteraceae* family widely used for inulin production in northwestern Europe, India, South Africa, and Chile (Street et al., 2013). Its roots are also processed to prepare dried or roasted products or beverages. Chicory, an important crop in the region Nord-Pas-de-Calais, France, accumulates four major polyphenols: chlorogenic, isochlorogenic, caftaric, and chicoric acids (Carazzone et al., 2013). These caffeic esters have been described for their antioxidant properties and also for potential therapeutic properties such as antidiabetic properties (Pushparaj et al., 2007; Tousch et al., 2008; Heimler et al., 2009). The biochemical pathways involved in the synthesis of these high-value bioactive compounds are far from being fully understood. Nevertheless, biochemical data indicate that they all arise from the phenylpropanoid pathway. Furthermore the precise regulation mechanisms controlling the synthesis of the four compounds highly interconnected at the metabolic level still need to be described. To get more insight into these pathways, high throughput gene expression analyses should be considered, and assessed by fluorescence-based quantitative real-time PCR (qRT-PCR) as a method of choice for accurate quantification of gene expression. Nevertheless several critical steps have to be followed in order to provide meaningful data. In particular identification of reference genes is a critical step in the design of qRT-PCR experiments. They are crucial for data normalization and will determine the reliability of the data and of their interpretation (Bustin et al., 2009). Good reference genes are stably expressed under chosen experimental conditions and should have a level of expression comparable to those of the target genes (Gimeno et al., 2014; Huang et al., 2014). The key difference between a reference gene and a real housekeeping gene is that a housekeeping gene should be stably expressed whatever the conditions, at every stage of the plant life, in every tissues. If such a gene existed, then no validation would be required. However, some genes as genes encoding GADPH or actin were sometimes inappropriately referred to as housekeeping gene. For example, *GADPH* is unstably expressed in papaya during storage at different temperature or under nitrogen stress in tomato and *actin* is unstably expressed under salinity stress in potato or in cucumber (Nicot et al., 2005; Løvdal and Lillo, 2009; Wan et al., 2010; Zhu et al., 2012). Therefore, it appears more appropriate to talk about reference genes rather than housekeeping genes.

In an attempt to decipher the phenylpropanoid pathway in chicory, we have established cell cultures from chicory roots. Due to their genetic stability and to their well-controlled culture conditions, cell cultures are a suitable model for secondary metabolism investigation (Collin, 2001; Hussain et al., 2012). Although reference genes have already been described for chicory plant, none has been assessed for cell suspension cultures (Maroufi et al., 2010). As a proof of principle, Methyl jasmonate (MeJA), known as an inducer of jasmonate metabolism and to regulate a set of physiological and developmental processes, was chosen to elicit cell suspension. Particularly, MeJA was shown to stimulate secondary metabolite production in plant cell cultures such as phenylpropanoid metabolism in *Nicotiana tabacum* cells (Gális et al., 2006). We have shown that chlorogenic and isochlorogenic acid accumulations were promoted by such a treatment in chicory cell cultures (unpublished results). Several studies have shed light on some mechanisms of the MeJA-elicited secondary metabolites biosynthesis (De Geyter et al., 2012; Wasternack and Hause, 2013). Nevertheless the detailed processes of MeJA stimulation of caffeic ester production and concomitant transcriptome changes associated with response to MeJA remain poorly understood.

In the current study, eighteen genes were tested as potential candidates for the normalization of qRT-PCR data from chicory cell cultures exposed to various conditions of elicitation. These genes were also tested on another study design, i.e., different organs of 6-week-old seedlings, in order to assess their potentiality under a variety of experimental conditions. Finally, 12 genes were ranked according to three of the most used softwares, i.e., geNorm, Normfinder and BestKeeper and reference genes suitable for data normalization were identified for both cell cultures and seedlings. Furthermore, the usefulness of these reference genes was tested by normalizing expression of some genes of interest (GOI) known to be induced by MeJA treatment in other plant species, i.e., *AOC, MYC2, C4H, PAL*, and *4CL*.

## Materials and Methods

### Plant Material

Experiments were conducted with *C. intybus* L. var *sativum* cv. Orchies. *C. intybus* plants were germinated from seeds originally received from the company Florimond Desprez (Cappelle en Pévèle, France). Cell suspension cultures of *C. intybus* were obtained from root explant derived callus. The cultures were maintained in a liquid Murashige and Skoog medium (MS) (Murashige and Skoog, 1962) containing sucrose (20 g.L^-1^), glutamine (500 mg.L^-1^), inositol (100 mg.L^-1^), naphthalene acetic acid (1.8 mg.L^-1^), kinetin (0.02 mg.L^-1^), and 2,4-dichlorophenoxyacetic acid (0.02 mg.L^-1^) in Erlenmeyer flasks on orbital shaker at an agitation speed of 110 rpm with a light source providing about 5 W.m^-2^ on a 12/12-h light/dark cycle with a temperature of 24 ± 1°C in the light phase and 20 ± 1°C in the dark phase. Elicitation experiments were conducted in triplicates on 20 mL subcultures seeded with about 1 g of fresh cells in 50 mL Erlenmeyer flasks. Then, MeJA dissolved in ethanol was added to a final concentration of 50 μM to elicit cells. As negative controls, cells kept in the reference medium alone or with ethanol added were used. Cells were harvested 24 h after the beginning of the experiment by vacuum filtration and rinsed with MS medium. Collected cells were immediately frozen in liquid nitrogen and stored at -80°C. Cells harvested before elicitation constitute another control. For experiments with seedlings, seeds of *C. intybus* were sown on filter paper impregnated with liquid MS medium 0.5X for 7 days (Murashige and Skoog, 1962). Seedlings were then transferred and grown hydroponically on the same medium for 6 weeks in a greenhouse on a 16/8-h light/dark cycle with a temperature of 24 ± 1°C in the light phase and 18 ± 1°C in the dark phase. Then, three young plants were individually dissected and sampled in four parts: root and leaves sorted by age. At this stage, six to eight leaves have emerged and so, leaves were separated in three equivalent groups, i.e., oldest leaves, middle-age leaves, and the remaining partially expanded leaves. The samples were immediately frozen in liquid nitrogen and stored at -80°C until use.

### RNA Extraction and cDNA Synthesis

Total RNA was extracted from cell cultures using Tri-Reagent (MRC, Inc) according to the manufacturer’s instructions. To avoid genomic DNA contamination, DNase treatment was performed using the Turbo DNA-Free Kit (Applied Biosystem). Total RNA from seedlings tissues was extracted using the Nucleospin RNA Kit (Macherey–Nagel) including a DNAse treatment. For both cell cultures and seedlings, RNA concentration, quality, and purity were determined using the Experion Automated Electrophoresis System (Bio-rad). cDNA was synthesized from 1 μg of total RNA with the Reverse Transcriptase Superscript III RNase H kit (Invitrogen) and oligo(dT)_20_ primer (Invitrogen), according to the manufacturer’s instruction in a final volume of 21 μL.

### Primer Design

The qRT-PCR primers were designed using Primer 3 with the following parameters: Tm between 59 and 61°C (optimum Tm of 60°C); 18–25 base pairs (bp) in length with the optimum length at 20 bp; GC content of 40–60% and a PCR product size between 60 and 150 bp. To check the primer’s specificity, conventional PCR was performed and the products analyzed by electrophoresis on 1.0% agarose gels stained using GelRed (Biotium). For both candidate reference genes and GOI, chicory sequences were retrieved after performing BLASTX searches with orthologous gene sequences of *Arabidopsis* as template. The database used was an expressed sequence tag (EST) database of a cDNA library derived from chicory roots produced using the Roche 454 sequencing platform.

### qRT-PCR Conditions and Analysis

The qRT-PCR were carried out in 96-wells plates with the iCycler iQ real time PCR detection system (Bio-Rad) using iQ SYBR Green Supermix (Bio-Rad) in a reaction volume of 20 μL containing 10 μL 2X iQ SYBR Green Supermix, 5 μL qRT-PCR template diluted appropriately and 1.5 μL of primer (5 μM). The cycling conditions were as recommended by the manufacturer: 3 min at 95°C preceding 40 cycles (10 s at 95°C followed by 30 s at 60°C). At the end of the run, a melting curve was generated by heating the amplicon from 60 to 95°C in order to confirm the specificity of the amplification for each primer pair. To check the absence of contamination by genomic DNA, PCR reactions on RNA templates were performed for each primer pair. All qRT-PCR were run in technical duplicates and biological triplicates. Standard curves were generated to calculate the PCR efficiency using a fivefold dilution series of a pooled sample containing an equal fraction of all cDNAs of the experiment. Efficiency (E) of each primer pair was obtained from the slope of the calibration curve generated according to the equation:

$$E=\left(10^{\frac{-1}{\mathrm{slope}}}-1\right)\times 100(Bustin et al., 2009).$$

Primers associated with amplification efficiency below 90% and above 110% were not considered.

### Statistical Analysis of the Reference Gene Expression Stability

Expression levels were determined as the number of cycles needed for the fluorescent signal to reach a threshold fixed in the exponential phase of the PCR reaction (Ct). Three different statistical algorithms were used to evaluate the stability of the reference genes: geNorm (Vandesompele et al., 2002), NormFinder (Andersen et al., 2004), and BestKeeper (Pfaﬄ et al., 2004). Ct values were converted into correct input files according to the requirement of the softwares.

### Determination of the Expression Profile of GOI

In order to validate the reliability of the selected reference genes, relative quantification (Q) of some genes known to be induced by MeJA was realized. The expression levels were calculated according to the Pffafl equation:

$$Q=\frac{{\left(E_{\mathrm{target}}\right)}^{\Delta \mathrm{Ct}}{{}_{\mathrm{target}}}^{\left(control-sample\right)}}{{\left(E_{\mathrm{ref}}\right)}^{\Delta \mathrm{Ct}}{{}_{\mathrm{ref}}}^{\left(control-sample\right)}}\;(Pfaffl, 2001).$$

E_target_ is the real-time PCR efficiency of target gene transcript; E_ref_ is the real-time PCR efficiency of reference gene transcript; ΔCt is the Ct deviation of control minus sample of the gene transcript.

ANalysis Of VAriance were conducted with the R software with a cut-off value of 0.05 (R Core Team, 2014).

## Results

### Selection of Candidate Reference Genes, Design of Primers, and Evaluation of Primer Specificity and PCR Efficiency

Eighteen candidate genes were chosen according to described *Arabidopsis* most stable genes and reference genes commonly used in qRT-PCR studies (Czechowski et al., 2005). Orthologous chicory sequences were retrieved after performing BLASTX on a chicory root EST database. These eighteen genes listed in **Table 1** were named according to *Arabidopsis* orthologs. The putative reference genes encode proteins with a wide variety of biological functions such as transcription factors, ubiquitous enzymes or cytoskeleton elements. The primer sequences, amplicon melting temperature (Tm) and length and PCR efficiencies are indicated in **Table 2**.

**Table 1:** Description of candidate reference genes, genes of interest (GOI), and comparison with *Arabidopsis* orthologs.

Gene abbreviation	Accession number	Gene description	*Arabidopsis* ortholog	*Arabidopsis* blastX *E*-value
*UBQ10*	KP752078	Ubiquitin 10	At4g05320	6.00E-150
*EF1α*	KP752079	Elongation Factor 1-α	At5g60390	0
*GAPDH*	DT210806.1	Glyceraldehyde 3-phosphate dehydrogenase	At1g13440	7.00E-172
*UBC9*	FL679405.1	Ubiquitin conjugating enzyme 9	At4g27960	7.00E-84
*ACT2*	KP752080	Actin 2	At3g18780	1.00E-31
*TIP41*	EH681285.1	TIP41 like protein	At4g34270	9.00E-105
*UBC*	EH709287.1	Ubiquitin-conjugating enzyme	At5g25760	8.00E-88
*PP2AA3*	EH675923.1	Protein phosphatase 2A subunit A3	At1g13320	1.00E-48
*bHLH*	KP752081	Transcription factor bHLH	At4g38070	5.00E-29
*CYP5*	EH688850.1	Cyclophilin 5	At2g29960	3.00E-78
*PROF*	DT2111339.1	Profilin	At2g19760	5.00E-57
*TUBβ9*	KP752082	Tubulin β-9	At4g20890	8.00E-87
*SAND*	KP752083	SAND family protein	At2g28390	0
*Clath*	EH684142.1	Clathrin adaptor complex subunit	At5g46630	0
*ACT7*	EH674624.1	Actin 7	At5g09810	0
*PP2AA2*	FL675704.1	Protein phosphatase 2A subunit A2	At3g25800	0
*αTUB*	EH691312.1	Tubulin α	At5g19780	0
*βTUB*	KP752084	Tubulin β-3	At5g62700	0
*AOC*	EH704455.1	Allene oxide cyclase	At1g13280	4.00E-60
*MYC2*	KP752085	Transcription factor MYC2	At1g32640	3.00E-150
*PAL*	KP752086	Phe ammonia-lyase	At2g37040	0
*C4H*	KP752087	Cinnamate 4-hydroxylase	At2g30490	0
*4CL*	EH704637.1	4-hydroxycinnamoyl-CoA ligase	At1g51680	0

**Table 2:** Primer sequences used for amplification of reference genes and GOI, amplicon length, melting temperature, and PCR efficiency.

Gene abbreviation	Primer sequence (forward/reverse primer)	Amplicon length	Tm	Efficiency
*ACT7*	5′-AGACAACACGGCCTGGATAG-3′5′-TGAAGAGCACCCGGTTTTAC-3′	130 bp	81°C	104%
*PROF*	5′-CCCAATTTCCCTCAGTTGAA-3′5′-TTGGTCCCACCACAGTGTAA-3′	104 bp	81°C	83.5%
*PP2AA3*	5′-TGCTTACCCTAGTGCCTCTGA-3′5′-TTCCCAAATTTGTAGCAGCA-3′	113 bp	80°C	96.2%
*GAPDH*	5′-GGTGAGAAACCCGTCACTGT-3′5′-CTTTGCACCACCCTTCAAGT-3′	104 bp	85.5°C	82.5%
*αTUB*	5′-GCACTTACCGCCAACTCTTC-3′5′-TTACTCGGTCAAGGCAAAGG-3′	126 bp	82.5°C	86.8%
*βTUB*	5′-TTTCCCGGTCAACTCAACTC-3′5′-ACTGTGAGGGCTCGGTATTG-3′	134 bp	85.5°C	95.5%
*Clath*	5′-TGCTTCCGCCATCTACTTTT-3′5′-TCCCAAGTTCCTTTGTTTGC-3′	128 bp	80.5°C	91%
*CYP5*	5′-CAGTGCCAAAAACAGCAGAA-3′5′-GGTGAAGTCACCTCCCTGAA-3′	143 bp	82°C	92.4%
*EF1α*	5′-AAGCCAGGTATGGTCGTCAC-3′5′-GACATTGTCTCCGGGAAGAG-3′	108 bp	85.5°C	96.2%
*PP2AA2*	5′-CATGGGCTCAGAAATCACCT-3′5′-ATTGGTCAACGATGGGGATA-3′	128 bp	82.5°C	93.9%
*SAND*	5’-TGCTTACACCACAAGGCAAG-3′5′-GAAGCAGCATGTCATCAGGA-3′	149 bp	82°C	104.9%
*TIP41*	5′-GTTGGGTGCGCATCTCTAAT-3′5′-AGCTCCGGCAGCTTTTACTT-3′	100 bp	83.5°C	93.2%
*TUB ß9*	5′-GCGATTCATTGCAAGGTTTT-3′5′-CATCATTCGATCGGGGTATT-3′	113 bp	84°C	98.4%
*UBC*	5′-GGTGGAGTTTTTCAGCTTGC-3′5′-TCAAGGCAAATCTCTCCTGTC-3′	125 bp	79.5°C	99.1%
*ACT2*	5′-AGGATCTTCAGCCCCTTGTT-3′5′-ACCATTGTCTGGCAGCCTAC-3′	119 bp	84°C	102%
*bHLH*	5′-GCAAAGTGGCTAAAGCTTCC-3′5′-CATGGGCTTCTTCCAAGTGT-3′	146 bp	79.5°C	107%
*UBQ10*	5′-GCGTTTGTTCATTGCTTCAA-3′5′-ATGGTGTCCGAGATTTCCAC-3′	177 bp	81°C	99.3%
*UBC9*	5′-CGGTCCTGTAGCGGAAGATA-3′5′-AGTCCTGAATGCAACCTTGG-3′	145 bp	82.5°C	90%
*AOC*	5′-CGACAGGAGGTTAGGAGCTG-3′5′-CGCATCCAGTAGCTTCATCA-3′	115 bp	86°C	101%
*MYC2*	5′-GGCTAATGGCGGTAATGAAA-3′5′-GTCAAGGCTAATCGGAGCTG-3′	142 bp	83.5°C	90%
*PAL*	5′-CATGGACAACACTCGTTTGG-3′5′-TTACGAGCTCGGAGAATTGG-3′	71 bp	82°C	96.6%
*C4H*	5′-CAAGCTTCCACCTGGACCTA-3′5′-GCCCATACGGAGAAGCAATA-3′	130 bp	85°C	90.5%
*4CL*	5′-ATCAATGAGCCCGATGTCTC-3′5′-CTTAACGATCCGGAAGCAAC-3′	75 bp	84°C	90%

*The genes discarded from the stability analyses are underlined. Gene abbreviations are explained in **Table 1**.*

Twelve candidate reference genes were selected on the basis of the amplification efficiency and primer specificity but also on the basis of their mean Ct value. Indeed, it’s preferable to use reference gene with a Ct value similar to Ct value of the GOI. Thus, genes with a too low or too high Ct value in our conditions like *EF1α* (mean Ct of 14) or *TUB β9* (mean Ct of 31) were discarded even if they are often used as reference genes.

### Expression Profile of the Candidate Reference Genes

**FIGURE 1:**
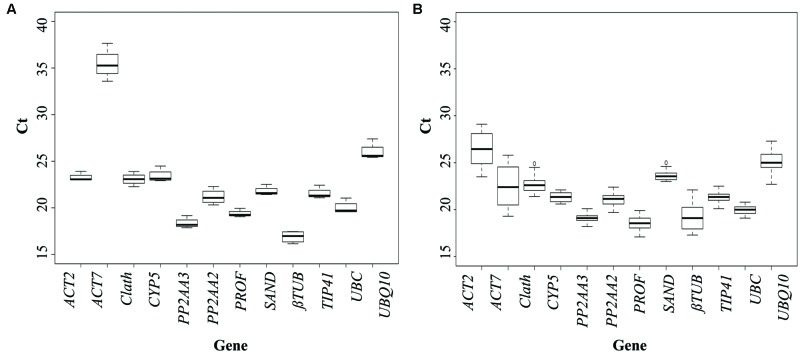
**Cycle threshold (Ct) values of the candidate reference genes across the different experimental samples (**A**: cell cultures; **B**: seedlings).** Box-plot graph of Ct values show the median values as line across the box. Lower and upper boxes indicating the first and the third quartile. Whiskers represent the maximum and minimum values.

To evaluate the expression stability of each candidate reference gene, Ct values were measured for each individual sample. The expression profiles of the genes across the cell cultures under different conditions of elicitation and across the different tissues of seedlings (see Materials and Methods for details) are shown in **Figure 1**. In cell cultures, all the tested genes are stably expressed except *ACT7*, which is also the gene with the highest mean Ct value. Regarding expression of the 12 potential reference genes in the seedlings, Ct values showed more significant variations than for cell cultures. This could be explained by a wider genetic variability for the seedlings than for the cell cultures. For seedlings, three genes seem to be really unstably expressed in the different organs: *ACT2, ACT7*, and *βTUB* so they probably wouldn’t be acceptable as reference genes.

### Reference Gene Stability Analysis

In order to rank the reference genes under our experimental conditions, three of the most popular algorithms were used: geNorm, NormFinder, and BestKeeper. geNorm is used to rank the genes in accordance to their expression stability value (M), which is the average of the pairwise variation of a particular gene with all other candidate reference genes. The lowest the *M* value is, the most stable the gene is. NormFinder provides a stability value as output for each gene, once again, the lower this value is, the higher the stability of the gene is. BestKeeper is used to rank the candidate reference genes according to their coefficient of correlation (r) to the BestKeeper Index, which has to be as close as possible to 1. For geNorm and Normfinder analysis, the 12 genes previously selected were ranked. BestKeeper algorithm authorized the simultaneous analysis of only 10 genes so the two least stable genes as reported by geNorm and NormFinder were removed from the analysis.

#### Reference Gene Stability in Root Cell Cultures

**FIGURE 2:**
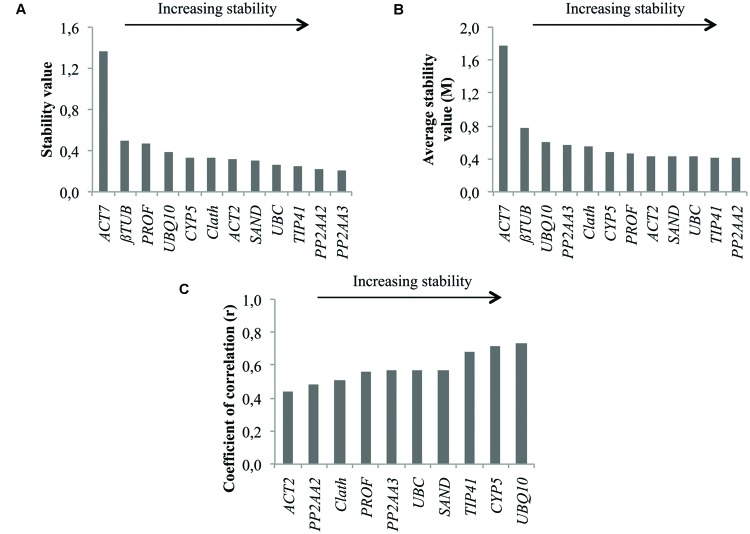
**Ranking of the candidate reference genes for chicory cell cultures under various conditions of elicitation according to NormFinder **(A)**, geNorm **(B)**, and BestKeeper (C)**.

In **Figure 2** are presented the rankings of the reference genes for root cell cultures of chicory obtained with the different algorithms. In agreement with the expression profiles presented in **Figure 1**, *ACT7* is the gene identified as the least stable by both geNorm and NormFinder. *βTUB* is the second least stable gene so these two genes were excluded from the BestKeeper analysis. For the other genes, NormFinder and GeNorm rankings are quite similar. Nevertheless, *PP2AA3*, which is the best gene according to geNorm is ranked ninth by NormFinder. Greater variations are observed with the BestKeeper ranking as often reported in previous published works (Wan et al., 2010; Xu et al., 2011). The performance of each gene as a reference gene was evaluated using their stability ranking in all three tests (**Table 3**). The most stable gene identified is *TIP41* followed by *PP2AA2* and *UBC*.

**Table 3:** Overall ranking of the candidate reference genes for cell cultures and for seedlings after geNorm, NormFinder, and BestKeeper analysis.

	Cell cultures					Seedlings				
Gene	geNorm rank	Normfinder rank	BestKeeper rank	Total score	Overall rank	geNorm rank	Normfinder rank	BestKeeper rank	Total score	Overall rank
*TIP41*	3	2	3	8	**1**	4	6	6	16	**4**
*PP2AA2*	2	1	9	12	**2**	3	3	5	11	**3**
*UBC*	4	3	5	12	**2**	5	4	8	17	**5**
*SAND*	5	4	4	13	**3**	2	2	3	7	**2**
*PP2AA3*	1	9	6	16	**4**	7	7	7	21	**6**
*CYP5*	8	7	2	17	**5**	6	5	10	21	**6**
*UBQ10*	9	10	1	20	**6**	9	8	4	21	**6**
*ACT2*	6	5	10	21	**7**	11	11	/	33	/
*Clath*	7	8	8	23	**8**	1	1	1	3	**1**
*PROF*	10	6	7	23	**8**	8	9	9	26	**8**
*βTUB*	11	11	/	33	/	10	10	2	22	**7**
*ACT7*	12	12	/	36	/	12	12	/	35	/

*The two lowest ranked genes according to geNorm and Normfinder were excluded from the BestKeeper analysis.*

#### Reference Gene Stability in Seedling Tissues

**FIGURE 3:**
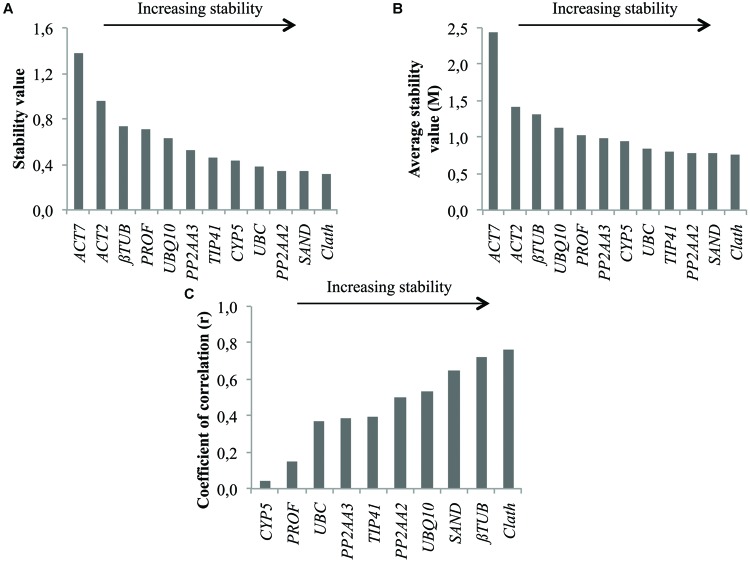
**Ranking of the candidate reference genes for different tissues of chicory seedlings according to NormFinder **(A),** geNorm **(B)**, and BestKeeper (C)**.

As for root cell cultures, stability of the 12 candidate reference genes was evaluated for seedling tissues and results are reported in **Figure 3**. The two least stable genes according to geNorm and NormFinder are the same as those identified on the basis of expression profiles, i.e., *ACT7* and *ACT2* (**Figure 1**). *βTUB* also exhibits wide variations of Ct and is indeed ranked as the third least stable gene by geNorm and NormFinder. However, BestKeeper surprisingly ranks β*TUB* as the second most stable gene. In all cases, *Clath* is the gene identified as the most suitable reference gene for normalization of qRT-PCR data from seedlings mRNA. *SAND* and *PP2AA2* are the next best according to their overall ranking (**Table 3**). It may also be noticed that this ranking of the 12 candidate reference genes is quite different from the one obtained for cell cultures.

**FIGURE 4:**
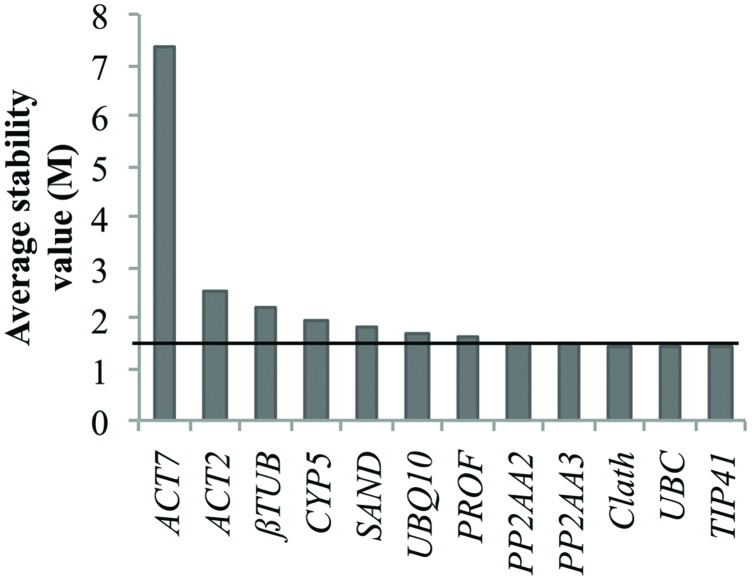
**Ranking of the candidate reference genes for both chicory seedlings and cell cultures according to geNorm.** The black line represents the threshold *M* value of 1.5 above which a putative reference gene is not considered as acceptable.

Nevertheless, we tried to identify reference genes suitable for both cell cultures and seedling expression profile analyses. The obtained results indicate *TIP41* as the most stably expressed gene over all the samples but the associated stability values were not good enough to be considered as acceptable for data normalization. Indeed, the geNorm stability analyze presented in **Figure 4** shows that only three of the candidate reference genes had a stability value slightly below 1.5 which is the threshold given by this algorithm (Vandesompele et al., 2002). Therefore, it might be better to conduct analyses of stability separately in order to keep the most suitable reference genes for each study design.

#### Determination of the Optimal Number of Reference Genes for Normalization

**FIGURE 5:**
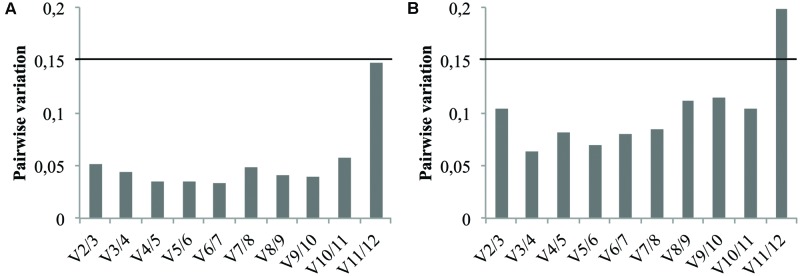
**Pairwise variation (Vn/Vn+1) analysis of the candidate reference genes for cell cultures **(A)** and for seedlings tissues **(B)**.** The black line represents the recommended threshold value of 0.15 below which the inclusion of an additional reference gene is not necessary.

In addition to the candidate reference gene ranking, the geNorm software can also be used to determine the optimal number of reference genes required for accurate normalization. This relies on calculation of the pairwise variation Vn/Vn+1. As recommended by Vandesompele et al. (2002) a Vn/Vn+1 value below the cutoff value of 0.15 means the addition of the *n*+1 reference gene is not required. Therefore, the first n references genes are sufficient to normalize qRT-PCR results. Consequently, pairwise variation values of the ranked reference genes were calculated for both cell cultures and seedlings tissues and the results are presented in **Figure 5**. In either case, the V2/3 value didn’t exceed the proposed cutoff value indicating that the use of the two most stable genes previously identified is sufficient for reliable normalization. It’s therefore recommended to use the combination of *TIP41* and *PP2AA2* (further referred as “*TIP41*+*PP2AA2*”) for normalization of cell cultures data while the combination of *Clath* and *SAND* are sufficient for analyzing data from seedlings tissues.

### Expression of GOI in Response to MeJA Induction

In order to validate the reliability of the reference genes previously selected for cell cultures, the relative quantification of five genes known to be induced by MeJA treatment was done. It consists of the genes encoding C4H (Cinnamate 4-hydroxylase), PAL (Phe ammonia-lyase), 4CL (4-hydroxycinnamoyl-CoA ligase), AOC (Allene oxide cyclase), and MYC2 (Transcription factor MYC2). *AOC* and *MYC2* are two genes directly involved in the jasmonic acid (JA) pathway. Indeed, *AOC* encodes an enzyme leading to the biosynthesis of JA whereas *MYC2* encodes a transcription factor that can activate transcription of early JA-responsive genes such as the one encoding MYC2 itself (Wasternack, 2007). These two genes have been shown to be induced by MeJA treatment in *Taxus chinensis* cell cultures (Li et al., 2012). C4H, PAL, and 4CL are three enzymes of the early phenylpropanoid pathway and their genes have been previously shown to be induced by MeJA treatment in *Nicotiana tabacum* cell cultures (Gális et al., 2006). *Arabidopsis* orthologs of these genes were identified by BLASTX and primers for qRT-PCR were designed accordingly (**Table 1**). Primers sequences, PCR efficiency and amplicon characteristics are summarized in **Table 2**.

**FIGURE 6:**
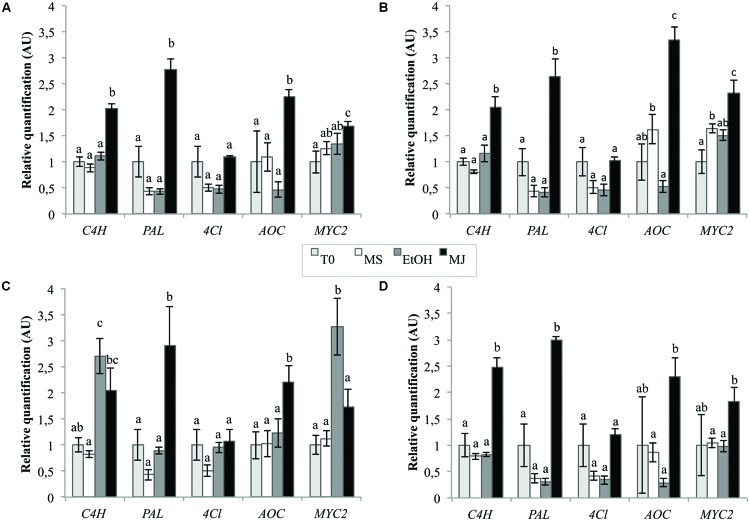
**Relative quantification of targeted genes expression in chicory cell cultures using different reference genes [TIP41 and PP2AA2 **(A)**, TIP41 alone **(B)**, βTUB **(C),** or Clath **(D)**] under various elicitation conditions.** EtOH: cell cultures treated with ethanol for 24 h, MJ: cell cultures treated with 50 μM MeJA for 24 h, MS: cultures kept on MS medium for 24 h, T0: cells sampled before elicitation. Error bars indicate SE of the mean. Different letters above histograms indicate statistical difference highlighted using ANOVA (*p*-value < 0.05).

The quantification was realized using normalization by the combination of the two most stable genes as previously determined and one of the least stables genes in cell cultures, i.e., “*TIP41*+*PP2AA2*” and *βTUB*, respectively. The best reference gene identified for seedlings, i.e., *Clath*, was also used to normalize the data as well as the best reference gene for cell cultures alone, i.e., *TIP41* to observe the consequence of the addition of another reference gene on the final results. The results presented in **Figure 6** show that the expression levels of *C4H, PAL, AOC*, and *MYC2* are significantly increased by the MeJA treatment (MJ) compare to the three negative controls, i.e., cell cultures treated with ethanol alone (EtOH), cell cultures without treatment (MS) or cell cultures before elicitation (T0). In the case of *4Cl*, no statistical differences were underlined within the different conditions. That said, it is nevertheless interesting to note that level of 4CL mRNA seems to be halved after 24 h of culture except when the medium is supplemented with MeJA. Anyway, relative quantifications obtained with normalization of the data by “*TIP41*+ *PP2AA2*” or by *TIP41* alone lead to similar trends. However, normalization by *βTUB* does not allow to highlight the increase in the expression levels of *MYC2, C4H*, and *AOC* under MeJA treatment compare to EtOH treatment. This normalization by *βTUB* even leads to conclusions opposite to those got with “*TIP41*+*PP2AA2*” as reference genes. On the other hand, normalization by *Clath* leads to similar expression pattern than normalization by the best references genes. Nevertheless, the results are more variable as shown by wider error bars and so, statistical differences are more difficult to underscore.

## Discussion

Our major research interests concern the study of the biochemical pathways involved in caffeic ester synthesis in chicory. In order to identify determinants of the phenylpropanoid pathways (effector and signaling components), genomic tools are useful and one of them is gene expression analysis by qRT-PCR. This technique is considered as the most appropriated method for gene expression profiling. It is due to its high sensitivity, specificity, accuracy and reproducibility. Nevertheless several criteria have to be strictly respected. In this purpose, validation of suitable reference genes for data normalization is mandatory to obtain reliable results. To our knowledge, only one previous work has reported the validation of reference genes in chicory (Maroufi et al., 2010). Nevertheless, this study of Maroufi et al. (2010) focused on old plants but we know that no universal reference gene can be found that would be suitable for all the different experimental designs. Thus, validation of specific reference genes is required for each design especially with models as different as old plant and cell cultures.

In this study, expression levels as well as stability of candidate reference genes were measured in chicory root cell cultures submitted to different conditions and also in different parts of chicory seedlings. Cell cultures were established and treated with MeJA as a known inducer of phenylpropanoid pathway. We have evaluated eighteen candidate reference genes in accordance with Remans et al. (2014) recommendation concerning the use of a starting pool of at least 10 reference genes for evaluation in new experimental conditions. Twelve of the initial eighteen genes were conserved for analyses with three different softwares: geNorm, NormFinder, and BestKeeper. Different rankings were obtained due to the different statistical algorithms associated. Therefore it is important to use at least three different softwares in order to achieve best results as possible in particular to avoid selection of co-regulated genes (Jacob et al., 2013). GeNorm, based on the pairwise variations with no consideration of a possible co-regulation, is particularly sensitive concerning this aspect (Huang et al., 2014). The 12 reference genes were ranked according to the results from the three algorithms.

In our experiment with cell cultures elicited with MeJA, *TIP41* is the most suitable gene among all the genes we have tested whereas for the study with different tissues of seedlings Clath was identified as the best reference gene. Rankings were significantly different between these two models. When analyzed together, one gene, *TIP41*, would be acceptable to normalize the data. Nevertheless, it is just slightly beyond the recommended geNorm threshold *M* value. Moreover classical reference genes such as the actin family genes, i.e., *ACT2* and *ACT7* didn’t perform well in the two considered cases whereas a gene encoding actin was ranked as the best in the previous study of Maroufi et al. (2010) concerning the validation of reference genes for chicory mature plants organs. This emphasizes the importance of validating the reference genes for each experimental design.

As a proof of principle, the reliability of this validation was evaluated by normalizing the expression of five genes reported to be induced by MeJA with the combination of the two top ranked genes, i.e., “*TIP41*+*PP2AA2*,” the best stable gene, i.e., *TIP41* or one of the least stable gene, i.e., β*TUB*. The results clearly show that normalization with inappropriately validated gene could lead to totally erroneous conclusions. Similarly, normalization with a reference gene identified as the best for the seedlings study design, i.e., *Clath* can not be used for the cell cultures study design to obtain reliable results. This constitutes another demonstration of the importance of a proper validation of reference genes each time the studied conditions vary. As a result, we could show that in chicory cells *MYC2, AOC, PAL*, and *C4H* expressions were induced by MeJA. *PAL, C4H*, and *4Cl* are core phenylpropanoid genes and were previously shown as co-induced through the action of a R2R3 MYB transcription factor, notably in tobacco cell culture (Gális et al., 2006). In our experimental conditions, no evidence of *4Cl* induction by MeJA has been given even if trends in this sense are observable. Selected 4Cl gene may not encode the isoform of this enzyme induced in these conditions. However, these results validate our experimental design and we can now use this system to try to identify new genes potentially involved in this metabolism.

## Conclusion

This study reports on the selection and validation of reference genes suitable for normalization of expression levels of GOI in chicory. We provide a list of reference genes as well as specific primers that may be used in the future by researchers working on *C. intybus*. The expression stability of 12 genes was evaluated across two different models, i.e., cell cultures elicited with MeJA and dissected seedlings using the three softwares geNorm, NormFinder, and BestKeeper. The most suitable reference genes were determined by comparing the results given by the three programs. Our data support the actual recommendations concerning a proper validation of reference genes for qRT-PCR each time the experimentation design changes even if based on the same plant.

## Conflict of Interest Statement

The authors declare that the research was conducted in the absence of any commercial or financial relationships that could be construed as a potential conflict of interest.
